# A mass accuracy sensitive probability based scoring algorithm for database searching of tandem mass spectrometry data

**DOI:** 10.1186/1471-2105-8-133

**Published:** 2007-04-20

**Authors:** Hua Xu, Michael A Freitas

**Affiliations:** 1 Department of Chemistry, the Ohio State University, Columbus 43210, OH, USA; 2 Department of Molecular Immunology Virology and Medical Genetics, the Ohio State University, Columbus 43210, OH, USA

## Abstract

**Background:**

Liquid chromatography coupled with tandem mass spectrometry (LC-MS/MS) has become one of the most used tools in mass spectrometry based proteomics. Various algorithms have since been developed to automate the process for modern high-throughput LC-MS/MS experiments.

**Results:**

A probability based statistical scoring model for assessing peptide and protein matches in tandem MS database search was derived. The statistical scores in the model represent the probability that a peptide match is a random occurrence based on the number or the total abundance of matched product ions in the experimental spectrum. The model also calculates probability based scores to assess protein matches. Thus the protein scores in the model reflect the significance of protein matches and can be used to differentiate true from random protein matches.

**Conclusion:**

The model is sensitive to high mass accuracy and implicitly takes mass accuracy into account during scoring. High mass accuracy will not only reduce false positives, but also improves the scores of true positive matches. The algorithm is incorporated in an automated database search program MassMatrix.

## Background

Liquid chromatography coupled with tandem mass spectrometry (LC-MS/MS) has become one of the most used tools in mass spectrometry based proteomics [1]. In shotgun proteomics, peptides are separated using liquid chromatography and introduced into a mass spectrometer via an ionization interface. In tandem mass spectrometry, the peptide precursor ions are isolated and fragmented via collision-induced dissociation (CID) [2] with inert gas, electron capture dissociation (ECD) [3], surface induced dissociation (SID) [4] and/or electron transfer dissociation (ETD) [5]. The resulting tandem MS spectra contain product ion signatures that relate back to the identity of the peptide precursor ions [2,6,7].

Various algorithms have since been developed to automate the process for modern high-throughput LC-MS/MS experiments. These algorithms fall under two categories: *de novo *sequence inference and database searching [8]. The first approach identifies peptide sequences directly from the tandem MS data [9,10]. This type of algorithm is usually computationally expensive and limited by the mass accuracy of the tandem MS data [8]. The database searching algorithms identify peptides by comparison with a protein sequence database [11]. In this approach, all potential peptides are created from the sequence database via digestion with proteases. Theoretical spectra containing product ion series appropriate for the given fragmentation technique are created for the peptides. All tandem MS spectra in the data set are then compared with the theoretical spectra [1]. Because of their relatively lower computation expense and higher compatibility with low mass accuracy spectra, database searching programs are more commonly used at this time [12].

There are also various probability based post-search methods used to statistically curate search results from database search algorithms [13,14]. These methods estimate the accuracy of protein/peptide identifications and compare search results from different algorithms based on a common standard. However, many models involve empirical parameters such as score from correlative scoring algorithms. Therefore they may possess biases as a result of parameter optimization or model training.

The key comparison between different algorithms lies in how each approach scores a potential match between experimental and theoretical spectra [11,15-25]. We recently developed a database searching program, MassMatrix that uses a mass accuracy sensitive statistical model for scoring. This approach is separate and distinct from algorithms that filter matches based on mass accuracy. In the latter high mass accuracy can be used to filter spectra by only searching tandem mass spectra whose precursor ion falls within the stated mass tolerance, and filtering product ions by high mass accuracy can further reduce the likelihood of a random match [26,27]. However, a score sensitive model implicitly takes mass accuracy into account during scoring. The model is rigorously derived and sensitive to the searching tolerance determined by the accuracy of mass spectrometer. High accuracy improves the sensitivity and selectivity of searches. The statistical scores represent the probability that a match is a random occurrence. In addition, a novel statistically derived algorithm to rigorously calculate protein scores from the statistically based peptide scores has been developed. Thus the protein scores reflect the significance of protein hits and can be used to differentiate true protein hits from random ones. Herein we describe the statistical models.

## Results

### Multiple scoring algorithms

The peptide matching algorithm contains two independent scoring models, including a descriptive model and a statistical model. These models are used to calculate three distinct scores for a peptide match. Each of the scores may be independently used to ascertain the quality of the match. Because each score is distinct, the combination of scores is useful for validating each peptide match. The two models and the application to calculating peptide match scores are described in detail in the following.

### Descriptive peptide scoring model

Descriptive scores do not strictly convey any statistical relevance and may be prone to bias due to the scoring parameters. However, they have proven to be useful and generally augment probability based scores [13]. The descriptive model used herein to calculate peptide match scores (S) is shown in eqn. 1.

S=100∑i=1nmatchIirmatch2max⁡(0,nmatch−3nmatch)Lpep
 MathType@MTEF@5@5@+=feaafiart1ev1aaatCvAUfKttLearuWrP9MDH5MBPbIqV92AaeXatLxBI9gBaebbnrfifHhDYfgasaacH8akY=wiFfYdH8Gipec8Eeeu0xXdbba9frFj0=OqFfea0dXdd9vqai=hGuQ8kuc9pgc9s8qqaq=dirpe0xb9q8qiLsFr0=vr0=vr0dc8meaabaqaciaacaGaaeqabaqabeGadaaakeaacqWGtbWucqGH9aqpcqaIXaqmcqaIWaamcqaIWaamdaWcaaqaamaaqahabaGaemysaK0aaSbaaSqaaiabdMgaPbqabaaabaGaemyAaKMaeyypa0JaeGymaedabaGaemOBa42aaSbaaWqaaiabb2gaTjabbggaHjabbsha0jabbogaJjabbIgaObqabaaaniabggHiLdGccqWGYbGCdaqhaaWcbaGaeeyBa0MaeeyyaeMaeeiDaqNaee4yamMaeeiAaGgabaGaeGOmaidaaOGagiyBa0MaeiyyaeMaeiiEaGNaeiikaGIaeGimaaJaeiilaWYaaSaaaeaacqWGUbGBdaWgaaWcbaGaeeyBa0MaeeyyaeMaeeiDaqNaee4yamMaeeiAaGgabeaakiabgkHiTiabiodaZaqaaiabd6gaUnaaBaaaleaacqqGTbqBcqqGHbqycqqG0baDcqqGJbWycqqGObaAaeqaaaaakiabcMcaPaqaamaakaaabaGaemitaW0aaSbaaSqaaiabbchaWjabbwgaLjabbchaWbqabaaabeaaaaaaaa@6B56@

*I*_*i *_is defined as the standardized abundance of the *i*^th ^product ion in the experimental spectrum (calculated by dividing the abundance of the *i*^th ^product ion by the maximum abundance in the spectrum), ∑i=1nmatchIi
 MathType@MTEF@5@5@+=feaafiart1ev1aaatCvAUfKttLearuWrP9MDH5MBPbIqV92AaeXatLxBI9gBaebbnrfifHhDYfgasaacH8akY=wiFfYdH8Gipec8Eeeu0xXdbba9frFj0=OqFfea0dXdd9vqai=hGuQ8kuc9pgc9s8qqaq=dirpe0xb9q8qiLsFr0=vr0=vr0dc8meaabaqaciaacaGaaeqabaqabeGadaaakeaadaaeWbqaaiabdMeajnaaBaaaleaacqWGPbqAaeqaaaqaaiabdMgaPjabg2da9iabigdaXaqaaiabd6gaUnaaBaaameaacqqGTbqBcqqGHbqycqqG0baDcqqGJbWycqqGObaAaeqaaaqdcqGHris5aaaa@3D25@ is the total standardized abundance of matched product ions, *n*_match _is the number of matched product ions, *r*_match _is the ratio of standardized abundance of matched product ions to total standardized abundance of the experimental spectrum, and *L*_pep _is the length of the peptide in the number of amino acids. Each of these factors contributes to the overall score as follows: ∑i=1nmatchIi
 MathType@MTEF@5@5@+=feaafiart1ev1aaatCvAUfKttLearuWrP9MDH5MBPbIqV92AaeXatLxBI9gBaebbnrfifHhDYfgasaacH8akY=wiFfYdH8Gipec8Eeeu0xXdbba9frFj0=OqFfea0dXdd9vqai=hGuQ8kuc9pgc9s8qqaq=dirpe0xb9q8qiLsFr0=vr0=vr0dc8meaabaqaciaacaGaaeqabaqabeGadaaakeaadaaeWbqaaiabdMeajnaaBaaaleaacqWGPbqAaeqaaaqaaiabdMgaPjabg2da9iabigdaXaqaaiabd6gaUnaaBaaameaacqqGTbqBcqqGHbqycqqG0baDcqqGJbWycqqGObaAaeqaaaqdcqGHris5aaaa@3D25@ evaluates the quality of the match, rmatch2
 MathType@MTEF@5@5@+=feaafiart1ev1aaatCvAUfKttLearuWrP9MDH5MBPbIqV92AaeXatLxBI9gBaebbnrfifHhDYfgasaacH8akY=wiFfYdH8Gipec8Eeeu0xXdbba9frFj0=OqFfea0dXdd9vqai=hGuQ8kuc9pgc9s8qqaq=dirpe0xb9q8qiLsFr0=vr0=vr0dc8meaabaqaciaacaGaaeqabaqabeGadaaakeaacqWGYbGCdaqhaaWcbaGaeeyBa0MaeeyyaeMaeeiDaqNaee4yamMaeeiAaGgabaGaeGOmaidaaaaa@35F5@ introduces a penalty for unmatched product ions, max⁡(0,nmatch−3nmatch)
 MathType@MTEF@5@5@+=feaafiart1ev1aaatCvAUfKttLearuWrP9MDH5MBPbIqV92AaeXatLxBI9gBaebbnrfifHhDYfgasaacH8akY=wiFfYdH8Gipec8Eeeu0xXdbba9frFj0=OqFfea0dXdd9vqai=hGuQ8kuc9pgc9s8qqaq=dirpe0xb9q8qiLsFr0=vr0=vr0dc8meaabaqaciaacaGaaeqabaqabeGadaaakeaacyGGTbqBcqGGHbqycqGG4baEcqGGOaakcqaIWaamcqGGSaaldaWcaaqaaiabd6gaUnaaBaaaleaacqqGTbqBcqqGHbqycqqG0baDcqqGJbWycqqGObaAaeqaaOGaeyOeI0IaeG4mamdabaGaemOBa42aaSbaaSqaaiabb2gaTjabbggaHjabbsha0jabbogaJjabbIgaObqabaaaaOGaeiykaKcaaa@46F3@ is an arbitrary penalty for matches with poor fragmentation, Lpep
 MathType@MTEF@5@5@+=feaafiart1ev1aaatCvAUfKttLearuWrP9MDH5MBPbIqV92AaeXatLxBI9gBaebbnrfifHhDYfgasaacH8akY=wiFfYdH8Gipec8Eeeu0xXdbba9frFj0=OqFfea0dXdd9vqai=hGuQ8kuc9pgc9s8qqaq=dirpe0xb9q8qiLsFr0=vr0=vr0dc8meaabaqaciaacaGaaeqabaqabeGadaaakeaadaGcaaqaaiabdYeamnaaBaaaleaacqqGWbaCcqqGLbqzcqqGWbaCaeqaaaqabaaaaa@3228@ is an additional penalty for peptides with long sequences and the constant 100 is used arbitrarily to scale the scores. By default, scores for a spectrum with less than three matched product ions will be 0 due to the arbitrary penalty. However, the minimum number of matched ions may be changed to any value. Reducing this number is especially valuable for the analysis of singly charged peptides that have characteristic C-terminal aspartic acid fragmentation [28]. The penalty for peptide length is included to normalize the scores. Peptides with longer sequences have more fragment ions and higher empirical scores than shorter sequences. The penalty results in long and short sequences both have similar scores for matches of similar quality. The choices of incorporating squared and square root for the terms *n*_match _and *L*_pep _were empirically determined from the evaluation of tandem MS data sets collected from LCQ and LTQ-Orbitrap mass spectrometers.

### Descriptive protein score

For "true" matches, we assume that the scores are normally distributed with a mean of 20 and a variance of 25. This arbitrary distribution estimates the distributions observed from analysis of several datasets. The expected contribution of each match to the protein score will be S×∫0Se−(x−20)2/5052πdx
 MathType@MTEF@5@5@+=feaafiart1ev1aaatCvAUfKttLearuWrP9MDH5MBPbIqV92AaeXatLxBI9gBaebbnrfifHhDYfgasaacH8akY=wiFfYdH8Gipec8Eeeu0xXdbba9frFj0=OqFfea0dXdd9vqai=hGuQ8kuc9pgc9s8qqaq=dirpe0xb9q8qiLsFr0=vr0=vr0dc8meaabaqaciaacaGaaeqabaqabeGadaaakeaacqWGtbWucqGHxdaTdaWdXaqaamaalaaabaGaemyzau2aaWbaaSqabeaacqGHsislcqGGOaakcqWG4baEcqGHsislcqaIYaGmcqaIWaamcqGGPaqkdaahaaadbeqaaiabikdaYaaaliabc+caViabiwda1iabicdaWaaaaOqaaiabiwda1maakaaabaGaeGOmaidcciGae8hWdahaleqaaaaaaeaacqaIWaamaeaacqWGtbWua0Gaey4kIipakiabdsgaKjabdIha4baa@4740@. Thus, the protein score from the descriptively scored matches is calculated from eqn. 2.

protein score=∑iSi×∫0Sie−(x−20)2/5052πdx
 MathType@MTEF@5@5@+=feaafiart1ev1aaatCvAUfKttLearuWrP9MDH5MBPbIqV92AaeXatLxBI9gBaebbnrfifHhDYfgasaacH8akY=wiFfYdH8Gipec8Eeeu0xXdbba9frFj0=OqFfea0dXdd9vqai=hGuQ8kuc9pgc9s8qqaq=dirpe0xb9q8qiLsFr0=vr0=vr0dc8meaabaqaciaacaGaaeqabaqabeGadaaakeaacqqGWbaCcqqGYbGCcqqGVbWBcqqG0baDcqqGLbqzcqqGPbqAcqqGUbGBcqqGGaaicqqGZbWCcqqGJbWycqqGVbWBcqqGYbGCcqqGLbqzcqGH9aqpdaaeqbqaaiabdofatnaaBaaaleaacqWGPbqAaeqaaOGaey41aq7aa8qmaeaadaWcaaqaaiabdwgaLnaaCaaaleqabaGaeyOeI0IaeiikaGIaemiEaGNaeyOeI0IaeGOmaiJaeGimaaJaeiykaKYaaWbaaWqabeaacqaIYaGmaaWccqGGVaWlcqaI1aqncqaIWaamaaaakeaacqaI1aqndaGcaaqaaiabikdaYiabec8aWbWcbeaaaaGccqWGKbazcqWG4baEaSqaaiabicdaWaqaaiabdofatnaaBaaameaacqWGPbqAaeqaaaqdcqGHRiI8aaWcbaGaemyAaKgabeqdcqGHris5aaaa@6036@

### Probability based peptide scoring model

In addition to the empirical score, a mass accuracy sensitive probability based scoring model was derived to evaluate peptide matches. The model determines the likelihood that an experimental spectrum match to a theoretical spectrum is a random occurrence. Consider a pair of spectra: one experimental and one theoretical. *W*_*e *_and *W*_*t *_denote their precursor masses respectively. In addition, the experimental data contains information regarding the abundance of product ions *I*_*i *_for each precursor, *W*_*e*_. The model ultimately tests the following two hypotheses: the null hypothesis, H_0, _states that a match is random, i.e. the theoretical spectrum is independent of the experimental; and the alternative hypothesis, H_A_, states that the match is not random, i.e. the theoretical spectrum is related to the experimental one.

Scoring the match is performed in two stages: 1) match *W*_*e *_against *W*_*t *_within the specified precursor ion mass accuracy and 2) match all product ions in the experimental spectrum against the theoretical within the specified mass accuracy. Both stages rely on calculating the probability that the occurrence of an ion within a fixed mass window could be a random occurrence (p=mass windowmass range
 MathType@MTEF@5@5@+=feaafiart1ev1aaatCvAUfKttLearuWrP9MDH5MBPbIqV92AaeXatLxBI9gBaebbnrfifHhDYfgasaacH8akY=wiFfYdH8Gipec8Eeeu0xXdbba9frFj0=OqFfea0dXdd9vqai=hGuQ8kuc9pgc9s8qqaq=dirpe0xb9q8qiLsFr0=vr0=vr0dc8meaabaqaciaacaGaaeqabaqabeGadaaakeaacqWGWbaCcqGH9aqpdaWcaaqaaiabb2gaTjabbggaHjabbohaZjabbohaZjabbccaGiabbEha3jabbMgaPjabb6gaUjabbsgaKjabb+gaVjabbEha3bqaaiabb2gaTjabbggaHjabbohaZjabbohaZjabbccaGiabbkhaYjabbggaHjabb6gaUjabbEgaNjabbwgaLbaaaaa@4AD8@).

To match the experimental precursor with that of theoretical peptide we first define the variable *q*:

q={1We and Wt match0otherwise
 MathType@MTEF@5@5@+=feaafiart1ev1aaatCvAUfKttLearuWrP9MDH5MBPbIqV92AaeXatLxBI9gBaebbnrfifHhDYfgasaacH8akY=wiFfYdH8Gipec8Eeeu0xXdbba9frFj0=OqFfea0dXdd9vqai=hGuQ8kuc9pgc9s8qqaq=dirpe0xb9q8qiLsFr0=vr0=vr0dc8meaabaqaciaacaGaaeqabaqabeGadaaakeaacqWGXbqCcqGH9aqpdaGabaqaauaabaqaciaaaeaacqaIXaqmaeaacqWGxbWvdaWgaaWcbaGaemyzaugabeaakiabbccaGiabbggaHjabb6gaUjabbsgaKjabbccaGiabdEfaxnaaBaaaleaacqWG0baDaeqaaOGaeeiiaaIaeeyBa0MaeeyyaeMaeeiDaqNaee4yamMaeeiAaGgabaGaeGimaadabaGaee4Ba8MaeeiDaqNaeeiAaGMaeeyzauMaeeOCaiNaee4DaCNaeeyAaKMaee4CamNaeeyzaugaaaGaay5Eaaaaaa@5142@

Under H_0_, the possibility that any precursor ion match (*q *= 1) could be random is given in eqn. 4.

p1=2τpepΠ
 MathType@MTEF@5@5@+=feaafiart1ev1aaatCvAUfKttLearuWrP9MDH5MBPbIqV92AaeXatLxBI9gBaebbnrfifHhDYfgasaacH8akY=wiFfYdH8Gipec8Eeeu0xXdbba9frFj0=OqFfea0dXdd9vqai=hGuQ8kuc9pgc9s8qqaq=dirpe0xb9q8qiLsFr0=vr0=vr0dc8meaabaqaciaacaGaaeqabaqabeGadaaakeaacqWGWbaCdaWgaaWcbaGaeGymaedabeaakiabg2da9maalaaabaGaeGOmaidcciGae8hXdq3aaSbaaSqaaiabbchaWjabbwgaLjabbchaWbqabaaakeaacqWFGoauaaaaaa@38DC@

In the above equation, *τ*_pep _is the mass accuracy of the precursor ion and *Π *is the detection range for the precursor ion. For each precursor ion the mass window is defined as ± *τ*_pep _(2 × *τ*_pep_). Thus *q *has a bernoulli (*p*_1_) distribution under H_0_. If the precursor ion masses of the pair of spectra do not match (*q *= 0) then the second stage is skipped. If *q *= 1 we proceed to stage 2 where we test the match of the experimental product ion spectrum against the theoretical spectrum.

The variable *b*_*i *_is defined for each product ion, *i*, in the experimental spectrum as follows:

bi={1peak i matches0otherwise.
 MathType@MTEF@5@5@+=feaafiart1ev1aaatCvAUfKttLearuWrP9MDH5MBPbIqV92AaeXatLxBI9gBaebbnrfifHhDYfgasaacH8akY=wiFfYdH8Gipec8Eeeu0xXdbba9frFj0=OqFfea0dXdd9vqai=hGuQ8kuc9pgc9s8qqaq=dirpe0xb9q8qiLsFr0=vr0=vr0dc8meaabaqaciaacaGaaeqabaqabeGadaaakeaacqWGIbGydaWgaaWcbaGaemyAaKgabeaakiabg2da9maaceaabaqbaeaabiGaaaqaaiabigdaXaqaaiabbchaWjabbwgaLjabbggaHjabbUgaRjabbccaGiabdMgaPjabbccaGiabb2gaTjabbggaHjabbsha0jabbogaJjabbIgaOjabbwgaLjabbohaZbqaaiabicdaWaqaaiabb+gaVjabbsha0jabbIgaOjabbwgaLjabbkhaYjabbEha3jabbMgaPjabbohaZjabbwgaLbaaaiaawUhaaiabc6caUaaa@52B0@

Under H_0_, all matched product ions are random and independent occurrences. The probability that a product ion randomly matches any of the product ions in the theoretical spectrum is:

p2=ΠtheoΠ
 MathType@MTEF@5@5@+=feaafiart1ev1aaatCvAUfKttLearuWrP9MDH5MBPbIqV92AaeXatLxBI9gBaebbnrfifHhDYfgasaacH8akY=wiFfYdH8Gipec8Eeeu0xXdbba9frFj0=OqFfea0dXdd9vqai=hGuQ8kuc9pgc9s8qqaq=dirpe0xb9q8qiLsFr0=vr0=vr0dc8meaabaqaciaacaGaaeqabaqabeGadaaakeaacqWGWbaCdaWgaaWcbaGaeGOmaidabeaakiabg2da9maalaaabaacciGae8hOda1aaSbaaSqaaiabbsha0jabbIgaOjabbwgaLjabb+gaVbqabaaakeaacqWFGoauaaaaaa@3901@

where Π_theo _is the total coverage of the detection range for all product ions in the theoretical spectrum and *Π *is the MS/MS detection range. It is assumed that *Π *is the same as the precursor ion mass range. However, for instruments that have a dynamic detection range assuming a fixed value *Π *will result in more conservative scores. For each product ion in the theoretical spectrum, the mass window is ± *τ*_msms _(2 × *τ*_msms_). If we assume there is no overlap in the product ion mass windows, then *Π*_theo _is calculated using the following equation

*Π*_theo _= 2 *m *× *τ*_msms_.

The probability that any single matched product ion (*b*_*i *_= 1) could be random can be calculated using the eqn. 8

p2=2m×τmsmsΠ
 MathType@MTEF@5@5@+=feaafiart1ev1aaatCvAUfKttLearuWrP9MDH5MBPbIqV92AaeXatLxBI9gBaebbnrfifHhDYfgasaacH8akY=wiFfYdH8Gipec8Eeeu0xXdbba9frFj0=OqFfea0dXdd9vqai=hGuQ8kuc9pgc9s8qqaq=dirpe0xb9q8qiLsFr0=vr0=vr0dc8meaabaqaciaacaGaaeqabaqabeGadaaakeaacqWGWbaCdaWgaaWcbaGaeGOmaidabeaakiabg2da9maalaaabaGaeGOmaiJaemyBa0Maey41aqlcciGae8hXdq3aaSbaaSqaaiabb2gaTjabbohaZjabb2gaTjabbohaZbqabaaakeaacqWFGoauaaaaaa@3DD5@

where *τ*_msms _is the product ion mass accuracy and *m *is the number of product ions within the detection range in the theoretical spectrum. Because the theoretical spectrum is independent of the experimental under H_0_, all *b*_*i *_(*i *= 1, 2 ..., n) are assumed to have an identical and independent bernoulli (*p*_2_) distribution under H_0_. The model is then used to perform two distinct tests. Each uses a different approach to evaluate the quality of a match: number of matched product ions *x *and total abundance of matched product ions *Y*.

### The pp score

The model is used to evaluate whether the number of matched product ions in an experimental spectrum could be a random occurrence. For all spectra whose precursor ion masses match, i.e. *q *= 1, the variable *x *is defined as the number of product ions in the experimental spectrum that match the theoretical spectrum (eqn. 9) where *b*_*i *_(*i *= 1, 2 ..., n) is defined in eqn. 5 and *n *is the number of product ions in the experimental spectrum.

x=∑i=1nbi
 MathType@MTEF@5@5@+=feaafiart1ev1aaatCvAUfKttLearuWrP9MDH5MBPbIqV92AaeXatLxBI9gBaebbnrfifHhDYfgasaacH8akY=wiFfYdH8Gipec8Eeeu0xXdbba9frFj0=OqFfea0dXdd9vqai=hGuQ8kuc9pgc9s8qqaq=dirpe0xb9q8qiLsFr0=vr0=vr0dc8meaabaqaciaacaGaaeqabaqabeGadaaakeaacqWG4baEcqGH9aqpdaaeWbqaaiabdkgaInaaBaaaleaacqWGPbqAaeqaaaqaaiabdMgaPjabg2da9iabigdaXaqaaiabd6gaUbqdcqGHris5aaaa@38EC@

Under H_0_, all *b*_*i *_have an identical and independent bernoulli (*p*_2_) distribution. Therefore, *x *will have a binomial (*n*, *p*_2_) distribution. Consequently the probability mass function for *x *is:

p(x)=n!x!(n−x)!p2x(1−p2)n−x
 MathType@MTEF@5@5@+=feaafiart1ev1aaatCvAUfKttLearuWrP9MDH5MBPbIqV92AaeXatLxBI9gBaebbnrfifHhDYfgasaacH8akY=wiFfYdH8Gipec8Eeeu0xXdbba9frFj0=OqFfea0dXdd9vqai=hGuQ8kuc9pgc9s8qqaq=dirpe0xb9q8qiLsFr0=vr0=vr0dc8meaabaqaciaacaGaaeqabaqabeGadaaakeaacqWGWbaCcqGGOaakcqWG4baEcqGGPaqkcqGH9aqpdaWcaaqaaiabd6gaUjabcgcaHaqaaiabdIha4jabcgcaHiabcIcaOiabd6gaUjabgkHiTiabdIha4jabcMcaPiabcgcaHaaacqWGWbaCdaqhaaWcbaGaeGOmaidabaGaemiEaGhaaOGaeiikaGIaeGymaeJaeyOeI0IaemiCaa3aaSbaaSqaaiabikdaYaqabaGccqGGPaqkdaahaaWcbeqaaiabd6gaUjabgkHiTiabdIha4baaaaa@4B32@

where *p*_2 _is calculated from eqn. 6. The p-value, *α*, is defined as the probability that the quality of a random match between a pair of spectra is greater than or equal to a match observed under H_0_. The pp value, *β*, is defined as the negative common logarithm of the p-value:

*β *= -log(*α*)

We use *x *to evaluate the quality of a match, such that the p-value is the probability that *x *for a random match between the pair of theoretical and experimental spectra is greater than or equal to that of the actual match, *x *= *n*_match_, under H_0_. The p-value is:

α=∑x=nmatchnp(x)=∑x=nmatchnn!x!(n−x)!p2x(1−p2)n−x
 MathType@MTEF@5@5@+=feaafiart1ev1aaatCvAUfKttLearuWrP9MDH5MBPbIqV92AaeXatLxBI9gBaebbnrfifHhDYfgasaacH8akY=wiFfYdH8Gipec8Eeeu0xXdbba9frFj0=OqFfea0dXdd9vqai=hGuQ8kuc9pgc9s8qqaq=dirpe0xb9q8qiLsFr0=vr0=vr0dc8meaabaqaciaacaGaaeqabaqabeGadaaakeaaiiGacqWFXoqycqGH9aqpdaaeWbqaaiabdchaWjabcIcaOiabdIha4jabcMcaPaWcbaGaemiEaGNaeyypa0JaemOBa42aaSbaaWqaaiabb2gaTjabbggaHjabbsha0jabbogaJjabbIgaObqabaaaleaacqWGUbGBa0GaeyyeIuoakiabg2da9maaqahabaWaaSaaaeaacqWGUbGBcqGGHaqiaeaacqWG4baEcqGGHaqicqGGOaakcqWGUbGBcqGHsislcqWG4baEcqGGPaqkcqGGHaqiaaaaleaacqWG4baEcqGH9aqpcqWGUbGBdaWgaaadbaGaeeyBa0MaeeyyaeMaeeiDaqNaee4yamMaeeiAaGgabeaaaSqaaiabd6gaUbqdcqGHris5aOGaemiCaa3aa0baaSqaaiabikdaYaqaaiabdIha4baakiabcIcaOiabigdaXiabgkHiTiabdchaWnaaBaaaleaacqaIYaGmaeqaaOGaeiykaKYaaWbaaSqabeaacqWGUbGBcqGHsislcqWG4baEaaaaaa@6AF2@

and the pp value is

β=−log⁡(α)=−log⁡(∑x=nmatchnn!x!(n−x)!p2x(1−p2)n−x)
 MathType@MTEF@5@5@+=feaafiart1ev1aaatCvAUfKttLearuWrP9MDH5MBPbIqV92AaeXatLxBI9gBaebbnrfifHhDYfgasaacH8akY=wiFfYdH8Gipec8Eeeu0xXdbba9frFj0=OqFfea0dXdd9vqai=hGuQ8kuc9pgc9s8qqaq=dirpe0xb9q8qiLsFr0=vr0=vr0dc8meaabaqaciaacaGaaeqabaqabeGadaaakeaaiiGacqWFYoGycqGH9aqpcqGHsislcyGGSbaBcqGGVbWBcqGGNbWzcqGGOaakcqWFXoqycqGGPaqkcqGH9aqpcqGHsislcyGGSbaBcqGGVbWBcqGGNbWzcqGGOaakdaaeWbqaamaalaaabaGaemOBa4MaeiyiaecabaGaemiEaGNaeiyiaeIaeiikaGIaemOBa4MaeyOeI0IaemiEaGNaeiykaKIaeiyiaecaaaWcbaGaemiEaGNaeyypa0JaemOBa42aaSbaaWqaaiabb2gaTjabbggaHjabbsha0jabbogaJjabbIgaObqabaaaleaacqWGUbGBa0GaeyyeIuoakiabdchaWnaaDaaaleaacqaIYaGmaeaacqWG4baEaaGccqGGOaakcqaIXaqmcqGHsislcqWGWbaCdaWgaaWcbaGaeGOmaidabeaakiabcMcaPmaaCaaaleqabaGaemOBa4MaeyOeI0IaemiEaGhaaOGaeiykaKcaaa@66F4@

### The pp2 score

The second approach evaluates whether the total abundance of matched product ions in the experimental spectrum could be a random occurrence. *Y *is defined as the total abundance of experimental product ions that match product ions in a given theoretical spectrum:

Y=∑i=1nIibi
 MathType@MTEF@5@5@+=feaafiart1ev1aaatCvAUfKttLearuWrP9MDH5MBPbIqV92AaeXatLxBI9gBaebbnrfifHhDYfgasaacH8akY=wiFfYdH8Gipec8Eeeu0xXdbba9frFj0=OqFfea0dXdd9vqai=hGuQ8kuc9pgc9s8qqaq=dirpe0xb9q8qiLsFr0=vr0=vr0dc8meaabaqaciaacaGaaeqabaqabeGadaaakeaacqWGzbqwcqGH9aqpdaaeWbqaaiabdMeajnaaBaaaleaacqWGPbqAaeqaaOGaemOyai2aaSbaaSqaaiabdMgaPbqabaaabaGaemyAaKMaeyypa0JaeGymaedabaGaemOBa4ganiabggHiLdaaaa@3B5A@

where *I*_*i *_is the standardized abundance of the *i*^th ^product ion in the experimental spectrum and *b*_*i *_is defined in eqn. 5. For clarity we define *y*_*i *_= *I*_*i *_*b*_*i *_to give eqn. 15.

Y=∑i=1nyi
 MathType@MTEF@5@5@+=feaafiart1ev1aaatCvAUfKttLearuWrP9MDH5MBPbIqV92AaeXatLxBI9gBaebbnrfifHhDYfgasaacH8akY=wiFfYdH8Gipec8Eeeu0xXdbba9frFj0=OqFfea0dXdd9vqai=hGuQ8kuc9pgc9s8qqaq=dirpe0xb9q8qiLsFr0=vr0=vr0dc8meaabaqaciaacaGaaeqabaqabeGadaaakeaacqWGzbqwcqGH9aqpdaaeWbqaaiabdMha5naaBaaaleaacqWGPbqAaeqaaaqaaiabdMgaPjabg2da9iabigdaXaqaaiabd6gaUbqdcqGHris5aaaa@38DC@

However, to complete the test we must know the inherent distribution of *Y*. This distribution is unknown and thus pp2 values can not be precisely calculated as were the pp values based on the total number of matched product ions. In order to estimate the pp2 value, three assumptions are needed:

1. *I*_*i *_is identically and independently distributed across product ions in the experimental spectrum,

2. *b*_*i *_is uncorrelated with *I*_*i *_in the experimental spectrum,

3. the number of product ions, *n*, in the experimental spectrum is large (*n *> 30).

Under assumption 1, the mean *μ*_*I *_and variance *σ*_*I*_^2 ^for the distribution of *I*_*i *_are estimated by:

{μ^I=1n∑i=1nIiσ^I2=1n−1∑i=1n(Ii−μ^I)2
 MathType@MTEF@5@5@+=feaafiart1ev1aaatCvAUfKttLearuWrP9MDH5MBPbIqV92AaeXatLxBI9gBaebbnrfifHhDYfgasaacH8akY=wiFfYdH8Gipec8Eeeu0xXdbba9frFj0=OqFfea0dXdd9vqai=hGuQ8kuc9pgc9s8qqaq=dirpe0xb9q8qiLsFr0=vr0=vr0dc8meaabaqaciaacaGaaeqabaqabeGadaaakeaadaGabaqaauaabaqaceaaaeaaiiGacuWF8oqBgaqcamaaBaaaleaacqWGjbqsaeqaaOGaeyypa0ZaaSaaaeaacqaIXaqmaeaacqWGUbGBaaWaaabCaeaacqWGjbqsdaWgaaWcbaGaemyAaKgabeaaaeaacqWGPbqAcqGH9aqpcqaIXaqmaeaacqWGUbGBa0GaeyyeIuoaaOqaaiqb=n8aZzaajaWaa0baaSqaaiabdMeajbqaaiabikdaYaaakiabg2da9maalaaabaGaeGymaedabaGaemOBa4MaeyOeI0IaeGymaedaamaaqahabaGaeiikaGIaemysaK0aaSbaaSqaaiabdMgaPbqabaGccqGHsislcuWF8oqBgaqcamaaBaaaleaacqWGjbqsaeqaaOGaeiykaKYaaWbaaSqabeaacqaIYaGmaaaabaGaemyAaKMaeyypa0JaeGymaedabaGaemOBa4ganiabggHiLdaaaaGccaGL7baaaaa@57C1@

Since *y*_*i *_= *I*_*i *_*b*_*i*_, assumption 2 yields eqn. 17 under H_0_,

{μy=Ey(yi)=EI(Ey|I(yi|Ii))=EI(p2Ii)=p2μIσy2=Ey(yi2)−Ey(yi)2=EI(Ey|I(yi2|Ii))−(p2μI)2=p2(1−p2)μI2+p2σ
 MathType@MTEF@5@5@+=feaafiart1ev1aaatCvAUfKttLearuWrP9MDH5MBPbIqV92AaeXatLxBI9gBaebbnrfifHhDYfgasaacH8akY=wiFfYdH8Gipec8Eeeu0xXdbba9frFj0=OqFfea0dXdd9vqai=hGuQ8kuc9pgc9s8qqaq=dirpe0xb9q8qiLsFr0=vr0=vr0dc8meaabaqaciaacaGaaeqabaqabeGadaaakeaadaGabaqaauaabaqaceaaaeaaiiGacqWF8oqBdaWgaaWcbaGaemyEaKhabeaakiabg2da9iabdweafnaaBaaaleaacqWG5bqEaeqaaOGaeiikaGIaemyEaK3aaSbaaSqaaiabdMgaPbqabaGccqGGPaqkcqGH9aqpcqWGfbqrdaWgaaWcbaGaemysaKeabeaakiabcIcaOiabdweafnaaBaaaleaacqWG5bqEcqGG8baFcqWGjbqsaeqaaOGaeiikaGIaemyEaK3aaSbaaSqaaiabdMgaPbqabaGccqGG8baFcqWGjbqsdaWgaaWcbaGaemyAaKgabeaakiabcMcaPiabcMcaPiabg2da9iabdweafnaaBaaaleaacqWGjbqsaeqaaOGaeiikaGIaemiCaa3aaSbaaSqaaiabikdaYaqabaGccqWGjbqsdaWgaaWcbaGaemyAaKgabeaakiabcMcaPiabg2da9iabdchaWnaaBaaaleaacqaIYaGmaeqaaOGae8hVd02aaSbaaSqaaiabdMeajbqabaaakeaacqWFdpWCdaqhaaWcbaGaemyEaKhabaGaeGOmaidaaOGaeyypa0Jaemyrau0aaSbaaSqaaiabdMha5bqabaGccqGGOaakcqWG5bqEdaqhaaWcbaGaemyAaKgabaGaeGOmaidaaOGaeiykaKIaeyOeI0Iaemyrau0aaSbaaSqaaiabdMha5bqabaGccqGGOaakcqWG5bqEdaWgaaWcbaGaemyAaKgabeaakiabcMcaPmaaCaaaleqabaGaeGOmaidaaOGaeyypa0Jaemyrau0aaSbaaSqaaiabdMeajbqabaGccqGGOaakcqWGfbqrdaWgaaWcbaGaemyEaKNaeiiFaWNaemysaKeabeaakiabcIcaOiabdMha5naaDaaaleaacqWGPbqAaeaacqaIYaGmaaGccqGG8baFcqWGjbqsdaWgaaWcbaGaemyAaKgabeaakiabcMcaPiabcMcaPiabgkHiTiabcIcaOiabdchaWnaaBaaaleaacqaIYaGmaeqaaOGae8hVd02aaSbaaSqaaiabdMeajbqabaGccqGGPaqkdaahaaWcbeqaaiabikdaYaaakiabg2da9iabdchaWnaaBaaaleaacqaIYaGmaeqaaOGaeiikaGIaeGymaeJaeyOeI0IaemiCaa3aaSbaaSqaaiabikdaYaqabaGccqGGPaqkcqWF8oqBdaqhaaWcbaGaemysaKeabaGaeGOmaidaaOGaey4kaSIaemiCaa3aaSbaaSqaaiabikdaYaqabaGccqWFdpWCaaaacaGL7baaaaa@A624@

Thus, *μ*_*y *_and *σ*_*y*_^2 ^can be estimated as:

{μ^y=p2μ^Iσ^y2=p2(1−p2)μ^I2+p2σ^I2
 MathType@MTEF@5@5@+=feaafiart1ev1aaatCvAUfKttLearuWrP9MDH5MBPbIqV92AaeXatLxBI9gBaebbnrfifHhDYfgasaacH8akY=wiFfYdH8Gipec8Eeeu0xXdbba9frFj0=OqFfea0dXdd9vqai=hGuQ8kuc9pgc9s8qqaq=dirpe0xb9q8qiLsFr0=vr0=vr0dc8meaabaqaciaacaGaaeqabaqabeGadaaakeaadaGabaqaauaabaqaceaaaeaaiiGacuWF8oqBgaqcamaaBaaaleaacqWG5bqEaeqaaOGaeyypa0JaemiCaa3aaSbaaSqaaiabikdaYaqabaGccuWF8oqBgaqcamaaBaaaleaacqWGjbqsaeqaaaGcbaGaf83WdmNbaKaadaqhaaWcbaGaemyEaKhabaGaeGOmaidaaOGaeyypa0JaemiCaa3aaSbaaSqaaiabikdaYaqabaGccqGGOaakcqaIXaqmcqGHsislcqWGWbaCdaWgaaWcbaGaeGOmaidabeaakiabcMcaPiqb=X7aTzaajaWaa0baaSqaaiabdMeajbqaaiabikdaYaaakiabgUcaRiabdchaWnaaBaaaleaacqaIYaGmaeqaaOGaf83WdmNbaKaadaqhaaWcbaGaemysaKeabaGaeGOmaidaaaaaaOGaay5Eaaaaaa@51AC@

According to the central limit theorem, *Y *is approximately a normal distribution with the following parameters under assumption 3, i.e. when *n *is large (*n *> 30)

{μY=nμyσY2=nσy2
 MathType@MTEF@5@5@+=feaafiart1ev1aaatCvAUfKttLearuWrP9MDH5MBPbIqV92AaeXatLxBI9gBaebbnrfifHhDYfgasaacH8akY=wiFfYdH8Gipec8Eeeu0xXdbba9frFj0=OqFfea0dXdd9vqai=hGuQ8kuc9pgc9s8qqaq=dirpe0xb9q8qiLsFr0=vr0=vr0dc8meaabaqaciaacaGaaeqabaqabeGadaaakeaadaGabaqaauaabaqaceaaaeaaiiGacqWF8oqBdaWgaaWcbaGaemywaKfabeaakiabg2da9iabd6gaUjab=X7aTnaaBaaaleaacqWG5bqEaeqaaaGcbaGae83Wdm3aa0baaSqaaiabdMfazbqaaiabikdaYaaakiabg2da9iabd6gaUjab=n8aZnaaDaaaleaacqWG5bqEaeaacqaIYaGmaaaaaaGccaGL7baaaaa@41BC@

The resulting probability density function is given in eqn. 20.

fY(Y)=e−(Y−μY)2/(2σY2)2πσY
 MathType@MTEF@5@5@+=feaafiart1ev1aaatCvAUfKttLearuWrP9MDH5MBPbIqV92AaeXatLxBI9gBaebbnrfifHhDYfgasaacH8akY=wiFfYdH8Gipec8Eeeu0xXdbba9frFj0=OqFfea0dXdd9vqai=hGuQ8kuc9pgc9s8qqaq=dirpe0xb9q8qiLsFr0=vr0=vr0dc8meaabaqaciaacaGaaeqabaqabeGadaaakeaacqWGMbGzdaWgaaWcbaGaemywaKfabeaakiabcIcaOiabdMfazjabcMcaPiabg2da9maalaaabaGaemyzau2aaWbaaSqabeaacqGHsislcqGGOaakcqWGzbqwcqGHsisliiGacqWF8oqBdaWgaaadbaGaemywaKfabeaaliabcMcaPmaaCaaameqabaGaeGOmaidaaSGaei4la8IaeiikaGIaeGOmaiJae83Wdm3aa0baaWqaaiabdMfazbqaaiabikdaYaaaliabcMcaPaaaaOqaamaakaaabaGaeGOmaiJae8hWdahaleqaaOGae83Wdm3aaSbaaSqaaiabdMfazbqabaaaaaaa@4BC3@

And *μ*_*Y *_and *σ*_*Y*_^2 ^are estimated by eqn. 21.

{μ^Y=n μ^y=n p2 μ^Iσ^Y2=n σ^y2=n {p2(1−p2)μ^I2+p2σ^I2}=n p2(1−p2)μ^I2+n p2σ^I2
 MathType@MTEF@5@5@+=feaafiart1ev1aaatCvAUfKttLearuWrP9MDH5MBPbIqV92AaeXatLxBI9gBaebbnrfifHhDYfgasaacH8akY=wiFfYdH8Gipec8Eeeu0xXdbba9frFj0=OqFfea0dXdd9vqai=hGuQ8kuc9pgc9s8qqaq=dirpe0xb9q8qiLsFr0=vr0=vr0dc8meaabaqaciaacaGaaeqabaqabeGadaaakeaadaGabaqaauaabaqaceaaaeaaiiGacuWF8oqBgaqcamaaBaaaleaacqWGzbqwaeqaaOGaeyypa0JaemOBa4MaeeiiaaIaf8hVd0MbaKaadaWgaaWcbaGaemyEaKhabeaakiabg2da9iabd6gaUjabbccaGiabdchaWnaaBaaaleaacqaIYaGmaeqaaOGaeeiiaaIaf8hVd0MbaKaadaWgaaWcbaGaemysaKeabeaaaOqaaiqb=n8aZzaajaWaa0baaSqaaiabdMfazbqaaiabikdaYaaakiabg2da9iabd6gaUjabbccaGiqb=n8aZzaajaWaa0baaSqaaiabdMha5bqaaiabikdaYaaakiabg2da9iabd6gaUjabbccaGiabcUha7jabdchaWnaaBaaaleaacqaIYaGmaeqaaOGaeiikaGIaeGymaeJaeyOeI0IaemiCaa3aaSbaaSqaaiabikdaYaqabaGccqGGPaqkcuWF8oqBgaqcamaaDaaaleaacqWGjbqsaeaacqaIYaGmaaGccqGHRaWkcqWGWbaCdaWgaaWcbaGaeGOmaidabeaakiqb=n8aZzaajaWaa0baaSqaaiabdMeajbqaaiabikdaYaaakiabc2ha9jabg2da9iabd6gaUjabbccaGiabdchaWnaaBaaaleaacqaIYaGmaeqaaOGaeiikaGIaeGymaeJaeyOeI0IaemiCaa3aaSbaaSqaaiabikdaYaqabaGccqGGPaqkcuWF8oqBgaqcamaaDaaaleaacqWGjbqsaeaacqaIYaGmaaGccqGHRaWkcqWGUbGBcqqGGaaicqWGWbaCdaWgaaWcbaGaeGOmaidabeaakiqb=n8aZzaajaWaa0baaSqaaiabdMeajbqaaiabikdaYaaaaaaakiaawUhaaaaa@812C@

The p-value, *α*, is the probability that *Y *for a random match is greater than or equal to that of the actual match, *I*_match_, under H_0_. The p-value becomes:

α=∫Imatch+∞fY(x)dx=∫Imatch+∞e−(x−μY)2/(2σY2)2πσYdx
 MathType@MTEF@5@5@+=feaafiart1ev1aaatCvAUfKttLearuWrP9MDH5MBPbIqV92AaeXatLxBI9gBaebbnrfifHhDYfgasaacH8akY=wiFfYdH8Gipec8Eeeu0xXdbba9frFj0=OqFfea0dXdd9vqai=hGuQ8kuc9pgc9s8qqaq=dirpe0xb9q8qiLsFr0=vr0=vr0dc8meaabaqaciaacaGaaeqabaqabeGadaaakeaaiiGacqWFXoqycqGH9aqpdaWdXaqaaiabdAgaMnaaBaaaleaacqWGzbqwaeqaaOGaeiikaGIaemiEaGNaeiykaKIaemizaqMaemiEaGhaleaacqWGjbqsdaWgaaadbaGaeeyBa0MaeeyyaeMaeeiDaqNaee4yamMaeeiAaGgabeaaaSqaaiabgUcaRiabg6HiLcqdcqGHRiI8aOGaeyypa0Zaa8qmaeaadaWcaaqaaiabdwgaLnaaCaaaleqabaGaeyOeI0IaeiikaGIaemiEaGNaeyOeI0Iae8hVd02aaSbaaWqaaiabdMfazbqabaWccqGGPaqkdaahaaadbeqaaiabikdaYaaaliabc+caViabcIcaOiabikdaYiab=n8aZnaaDaaameaacqWGzbqwaeaacqaIYaGmaaWccqGGPaqkaaaakeaadaGcaaqaaiabikdaYiab=b8aWbWcbeaakiab=n8aZnaaBaaaleaacqWGzbqwaeqaaaaakiabdsgaKjabdIha4bWcbaGaemysaK0aaSbaaWqaaiabb2gaTjabbggaHjabbsha0jabbogaJjabbIgaObqabaaaleaacqGHRaWkcqGHEisPa0Gaey4kIipaaaa@6D9F@

and is estimated by:

α=∫Imatch+∞e−(x−μ^Y)2/(2σ^Y2)2πσ^Ydx
 MathType@MTEF@5@5@+=feaafiart1ev1aaatCvAUfKttLearuWrP9MDH5MBPbIqV92AaeXatLxBI9gBaebbnrfifHhDYfgasaacH8akY=wiFfYdH8Gipec8Eeeu0xXdbba9frFj0=OqFfea0dXdd9vqai=hGuQ8kuc9pgc9s8qqaq=dirpe0xb9q8qiLsFr0=vr0=vr0dc8meaabaqaciaacaGaaeqabaqabeGadaaakeaaiiGacqWFXoqycqGH9aqpdaWdXaqaamaalaaabaGaemyzau2aaWbaaSqabeaacqGHsislcqGGOaakcqWG4baEcqGHsislcuWF8oqBgaqcamaaBaaameaacqWGzbqwaeqaaSGaeiykaKYaaWbaaWqabeaacqaIYaGmaaWccqGGVaWlcqGGOaakcqaIYaGmcuWFdpWCgaqcamaaDaaameaacqWGzbqwaeaacqaIYaGmaaWccqGGPaqkaaaakeaadaGcaaqaaiabikdaYiab=b8aWbWcbeaakiqb=n8aZzaajaWaaSbaaSqaaiabdMfazbqabaaaaOGaemizaqMaemiEaGhaleaacqWGjbqsdaWgaaadbaGaeeyBa0MaeeyyaeMaeeiDaqNaee4yamMaeeiAaGgabeaaaSqaaiabgUcaRiabg6HiLcqdcqGHRiI8aaaa@5778@

resulting in the pp2 value, *β*, as follows:

β=−log⁡(α)=−log⁡(∫Imatch+∞e−(x−μ^Y)2/(2σ^Y2)2πσ^Ydx)
 MathType@MTEF@5@5@+=feaafiart1ev1aaatCvAUfKttLearuWrP9MDH5MBPbIqV92AaeXatLxBI9gBaebbnrfifHhDYfgasaacH8akY=wiFfYdH8Gipec8Eeeu0xXdbba9frFj0=OqFfea0dXdd9vqai=hGuQ8kuc9pgc9s8qqaq=dirpe0xb9q8qiLsFr0=vr0=vr0dc8meaabaqaciaacaGaaeqabaqabeGadaaakeaaiiGacqWFYoGycqGH9aqpcqGHsislcyGGSbaBcqGGVbWBcqGGNbWzcqGGOaakcqWFXoqycqGGPaqkcqGH9aqpcqGHsislcyGGSbaBcqGGVbWBcqGGNbWzcqGGOaakdaWdXaqaamaalaaabaGaemyzau2aaWbaaSqabeaacqGHsislcqGGOaakcqWG4baEcqGHsislcuWF8oqBgaqcamaaBaaameaacqWGzbqwaeqaaSGaeiykaKYaaWbaaWqabeaacqaIYaGmaaWccqGGVaWlcqGGOaakcqaIYaGmcuWFdpWCgaqcamaaDaaameaacqWGzbqwaeaacqaIYaGmaaWccqGGPaqkaaaakeaadaGcaaqaaiabikdaYiab=b8aWbWcbeaakiqb=n8aZzaajaWaaSbaaSqaaiabdMfazbqabaaaaOGaemizaqMaemiEaGhaleaacqWGjbqsdaWgaaadbaGaeeyBa0MaeeyyaeMaeeiDaqNaee4yamMaeeiAaGgabeaaaSqaaiabgUcaRiabg6HiLcqdcqGHRiI8aOGaeiykaKcaaa@679E@

The pp2 value can be estimated by equation 17 very efficiently. However, the real distribution of *Y *is more tailed to larger values than the normal distribution. Therefore, pp2 values are overestimated when they are large.

### Distribution of pp value for random matches

When *q *= 0, the algorithm always assigns pp value, *β *= 0 because the experimental and theoretical precursor ions do not match. The cumulative distribution function for pp value when *q *= 0 is shown in eqn. 25.

Fβ|q=0(β)={1β=00β>0
 MathType@MTEF@5@5@+=feaafiart1ev1aaatCvAUfKttLearuWrP9MDH5MBPbIqV92AaeXatLxBI9gBaebbnrfifHhDYfgasaacH8akY=wiFfYdH8Gipec8Eeeu0xXdbba9frFj0=OqFfea0dXdd9vqai=hGuQ8kuc9pgc9s8qqaq=dirpe0xb9q8qiLsFr0=vr0=vr0dc8meaabaqaciaacaGaaeqabaqabeGadaaakeaacqWGgbGrdaWgaaWcbaacciGae8NSdiMaeiiFaWNaemyCaeNaeyypa0JaeGimaadabeaakiabcIcaOiab=j7aIjabcMcaPiabg2da9maaceaabaqbaeqabiGaaaqaaiabigdaXaqaaiab=j7aIjabg2da9iabicdaWaqaaiabicdaWaqaaiab=j7aIjabg6da+iabicdaWaaaaiaawUhaaaaa@42FC@

In statistical hypothesis testing, a p-value for a null hypothesis H_0 _is always a uniform distribution on the interval [0, 1]. Therefore, the cumulative distribution function for p-value of a random match is continuously distributed as

*F*_*α*|*q *= 1_(*α*) = *α*   (0 ≤ *α*≤ 1)

when *q *= 1. According to the definition of pp value (eqn. 11), the cumulative distribution function for pp value when *q *= 1 is

*F*_*β*|*q *= 1_(*β*) = 1 - 10^-*β*^   (*β *≥ 0)

and the probability density function is

fβ|q=1(β)=ddβFβ|q=1(β)=ddβ(1−10−β)=ln⁡(10)10−β(β≥0).
 MathType@MTEF@5@5@+=feaafiart1ev1aaatCvAUfKttLearuWrP9MDH5MBPbIqV92AaeXatLxBI9gBaebbnrfifHhDYfgasaacH8akY=wiFfYdH8Gipec8Eeeu0xXdbba9frFj0=OqFfea0dXdd9vqai=hGuQ8kuc9pgc9s8qqaq=dirpe0xb9q8qiLsFr0=vr0=vr0dc8meaabaqaciaacaGaaeqabaqabeGadaaakeaafaqabeqacaaabaGaemOzay2aaSbaaSqaaGGaciab=j7aIjabcYha8jabdghaXjabg2da9iabigdaXaqabaGccqGGOaakcqWFYoGycqGGPaqkcqGH9aqpdaWcaaqaaiabdsgaKbqaaiabdsgaKjab=j7aIbaacqWGgbGrdaWgaaWcbaGae8NSdiMaeiiFaWNaemyCaeNaeyypa0JaeGymaedabeaakiabcIcaOiab=j7aIjabcMcaPiabg2da9maalaaabaGaemizaqgabaGaemizaqMae8NSdigaaiabcIcaOiabigdaXiabgkHiTiabigdaXiabicdaWmaaCaaaleqabaGaeyOeI0Iae8NSdigaaOGaeiykaKIaeyypa0JagiiBaWMaeiOBa4MaeiikaGIaeGymaeJaeGimaaJaeiykaKIaeGymaeJaeGimaaZaaWbaaSqabeaacqGHsislcqWFYoGyaaaakeaacqGGOaakcqWFYoGycqGHLjYScqaIWaamcqGGPaqkaaGaeiOla4caaa@68E6@

Matches with pp or pp2 values under a critical value *β*_*c *_> 0 are discarded, i.e. their pp values are assigned 0. Thus the distribution of pp value for random matches returned by the algorithm is

Fβ|q=1∗(0)=∫0βcfβ|q=1(x)dx=∫0βcln⁡(10)10−xdx=1−10−βc
 MathType@MTEF@5@5@+=feaafiart1ev1aaatCvAUfKttLearuWrP9MDH5MBPbIqV92AaeXatLxBI9gBaebbnrfifHhDYfgasaacH8akY=wiFfYdH8Gipec8Eeeu0xXdbba9frFj0=OqFfea0dXdd9vqai=hGuQ8kuc9pgc9s8qqaq=dirpe0xb9q8qiLsFr0=vr0=vr0dc8meaabaqaciaacaGaaeqabaqabeGadaaakeaacqWGgbGrdaqhaaWcbaacciGae8NSdiMaeiiFaWNaemyCaeNaeyypa0JaeGymaedabaGaey4fIOcaaOGaeiikaGIaeGimaaJaeiykaKIaeyypa0Zaa8qmaeaacqWGMbGzdaWgaaWcbaGae8NSdiMaeiiFaWNaemyCaeNaeyypa0JaeGymaedabeaakiabcIcaOiabdIha4jabcMcaPiabdsgaKjabdIha4bWcbaGaeGimaadabaGae8NSdi2aaSbaaWqaaiabdogaJbqabaaaniabgUIiYdGccqGH9aqpdaWdXaqaaiGbcYgaSjabc6gaUjabcIcaOiabigdaXiabicdaWiabcMcaPiabigdaXiabicdaWmaaCaaaleqabaGaeyOeI0IaemiEaGhaaaqaaiabicdaWaqaaiab=j7aInaaBaaameaacqWGJbWyaeqaaaqdcqGHRiI8aOGaemizaqMaemiEaGNaeyypa0JaeGymaeJaeyOeI0IaeGymaeJaeGimaaZaaWbaaSqabeaacqGHsislcqWFYoGydaWgaaadbaGaem4yamgabeaaaaaaaa@6B04@

and for *β *≥ *β*_*c *_> 0,

Fβ|q=1∗(β)=Fβ|q=1(β)=1−10−β
 MathType@MTEF@5@5@+=feaafiart1ev1aaatCvAUfKttLearuWrP9MDH5MBPbIqV92AaeXatLxBI9gBaebbnrfifHhDYfgasaacH8akY=wiFfYdH8Gipec8Eeeu0xXdbba9frFj0=OqFfea0dXdd9vqai=hGuQ8kuc9pgc9s8qqaq=dirpe0xb9q8qiLsFr0=vr0=vr0dc8meaabaqaciaacaGaaeqabaqabeGadaaakeaacqWGgbGrdaqhaaWcbaacciGae8NSdiMaeiiFaWNaemyCaeNaeyypa0JaeGymaedabaGaey4fIOcaaOGaeiikaGIae8NSdiMaeiykaKIaeyypa0JaemOray0aaSbaaSqaaiab=j7aIjabcYha8jabdghaXjabg2da9iabigdaXaqabaGccqGGOaakcqWFYoGycqGGPaqkcqGH9aqpcqaIXaqmcqGHsislcqaIXaqmcqaIWaamdaahaaWcbeqaaiabgkHiTiab=j7aIbaaaaa@4C51@

Thus when *q *= 1

Fβ|q=1∗(β)={1−10−βcβ=01−10−ββ≥βc.
 MathType@MTEF@5@5@+=feaafiart1ev1aaatCvAUfKttLearuWrP9MDH5MBPbIqV92AaeXatLxBI9gBaebbnrfifHhDYfgasaacH8akY=wiFfYdH8Gipec8Eeeu0xXdbba9frFj0=OqFfea0dXdd9vqai=hGuQ8kuc9pgc9s8qqaq=dirpe0xb9q8qiLsFr0=vr0=vr0dc8meaabaqaciaacaGaaeqabaqabeGadaaakeaacqWGgbGrdaqhaaWcbaacciGae8NSdiMaeiiFaWNaemyCaeNaeyypa0JaeGymaedabaGaey4fIOcaaOGaeiikaGIae8NSdiMaeiykaKIaeyypa0ZaaiqaaeaafaqaaeGacaaabaGaeGymaeJaeyOeI0IaeGymaeJaeGimaaZaaWbaaSqabeaacqGHsislcqWFYoGydaWgaaadbaGaem4yamgabeaaaaaakeaacqWFYoGycqGH9aqpcqaIWaamaeaacqaIXaqmcqGHsislcqaIXaqmcqaIWaamdaahaaWcbeqaaiabgkHiTiab=j7aIbaaaOqaaiab=j7aIjabgwMiZkab=j7aInaaBaaaleaacqWGJbWyaeqaaaaaaOGaay5EaaGaeiOla4caaa@5456@

Likewise we can specify the unconditional distribution of pp values for random matches as follows. Since *q *has a bernoulli (*p*_1_) distribution, we have

P(q)={1−p1q=0p1q=1,
 MathType@MTEF@5@5@+=feaafiart1ev1aaatCvAUfKttLearuWrP9MDH5MBPbIqV92AaeXatLxBI9gBaebbnrfifHhDYfgasaacH8akY=wiFfYdH8Gipec8Eeeu0xXdbba9frFj0=OqFfea0dXdd9vqai=hGuQ8kuc9pgc9s8qqaq=dirpe0xb9q8qiLsFr0=vr0=vr0dc8meaabaqaciaacaGaaeqabaqabeGadaaakeaacqWGqbaucqGGOaakcqWGXbqCcqGGPaqkcqGH9aqpdaGabaqaauaabaqaciaaaeaacqaIXaqmcqGHsislcqWGWbaCdaWgaaWcbaGaeGymaedabeaaaOqaaiabdghaXjabg2da9iabicdaWaqaaiabdchaWnaaBaaaleaacqaIXaqmaeqaaaGcbaGaemyCaeNaeyypa0JaeGymaedaaaGaay5EaaGaeiilaWcaaa@41BC@

For *β *= 0 the cumulative distribution function becomes,

Fβ(0)=Fβ|q=0(0)×Pq(0)+Fβ|q=1∗(0)×Pq(1)=1×(1−p1)+(1−10−βc)×p1=1−p110−βc
 MathType@MTEF@5@5@+=feaafiart1ev1aaatCvAUfKttLearuWrP9MDH5MBPbIqV92AaeXatLxBI9gBaebbnrfifHhDYfgasaacH8akY=wiFfYdH8Gipec8Eeeu0xXdbba9frFj0=OqFfea0dXdd9vqai=hGuQ8kuc9pgc9s8qqaq=dirpe0xb9q8qiLsFr0=vr0=vr0dc8meaabaqaciaacaGaaeqabaqabeGadaaakeaafaqadeGabaaabaGaemOray0aaSbaaSqaaGGaciab=j7aIbqabaGccqGGOaakcqaIWaamcqGGPaqkcqGH9aqpcqWGgbGrdaWgaaWcbaGae8NSdiMaeiiFaWNaemyCaeNaeyypa0JaeGimaadabeaakiabcIcaOiabicdaWiabcMcaPiabgEna0kabdcfaqnaaBaaaleaacqWGXbqCaeqaaOGaeiikaGIaeGimaaJaeiykaKIaey4kaSIaemOray0aa0baaSqaaiab=j7aIjabcYha8jabdghaXjabg2da9iabigdaXaqaaiabgEHiQaaakiabcIcaOiabicdaWiabcMcaPiabgEna0kabdcfaqnaaBaaaleaacqWGXbqCaeqaaOGaeiikaGIaeGymaeJaeiykaKcabaGaeyypa0JaeGymaeJaey41aqRaeiikaGIaeGymaeJaeyOeI0IaemiCaa3aaSbaaSqaaiabigdaXaqabaGccqGGPaqkcqGHRaWkcqGGOaakcqaIXaqmcqGHsislcqaIXaqmcqaIWaamdaahaaWcbeqaaiabgkHiTiab=j7aInaaBaaameaacqWGJbWyaeqaaaaakiabcMcaPiabgEna0kabdchaWnaaBaaaleaacqaIXaqmaeqaaOGaeyypa0JaeGymaeJaeyOeI0IaemiCaa3aaSbaaSqaaiabigdaXaqabaGccqaIXaqmcqaIWaamdaahaaWcbeqaaiabgkHiTiab=j7aInaaBaaameaacqWGJbWyaeqaaaaaaaaaaa@7DD6@

and for *β *≥ *β*_*c *_> 0, it becomes,

Fβ(β)=Fβ|q=0(β)×Pq(0)+Fβ|q=1∗(β)×Pq(1)=0×(1−p1)+(1−10−β)×p1=1−p110−β
 MathType@MTEF@5@5@+=feaafiart1ev1aaatCvAUfKttLearuWrP9MDH5MBPbIqV92AaeXatLxBI9gBaebbnrfifHhDYfgasaacH8akY=wiFfYdH8Gipec8Eeeu0xXdbba9frFj0=OqFfea0dXdd9vqai=hGuQ8kuc9pgc9s8qqaq=dirpe0xb9q8qiLsFr0=vr0=vr0dc8meaabaqaciaacaGaaeqabaqabeGadaaakeaafaqadeGabaaabaGaemOray0aaSbaaSqaaGGaciab=j7aIbqabaGccqGGOaakcqWFYoGycqGGPaqkcqGH9aqpcqWGgbGrdaWgaaWcbaGae8NSdiMaeiiFaWNaemyCaeNaeyypa0JaeGimaadabeaakiabcIcaOiab=j7aIjabcMcaPiabgEna0kabdcfaqnaaBaaaleaacqWGXbqCaeqaaOGaeiikaGIaeGimaaJaeiykaKIaey4kaSIaemOray0aa0baaSqaaiab=j7aIjabcYha8jabdghaXjabg2da9iabigdaXaqaaiabgEHiQaaakiabcIcaOiab=j7aIjabcMcaPiabgEna0kabdcfaqnaaBaaaleaacqWGXbqCaeqaaOGaeiikaGIaeGymaeJaeiykaKcabaGaeyypa0JaeGimaaJaey41aqRaeiikaGIaeGymaeJaeyOeI0IaemiCaa3aaSbaaSqaaiabigdaXaqabaGccqGGPaqkcqGHRaWkcqGGOaakcqaIXaqmcqGHsislcqaIXaqmcqaIWaamdaahaaWcbeqaaiabgkHiTiab=j7aIbaakiabcMcaPiabgEna0kabdchaWnaaBaaaleaacqaIXaqmaeqaaOGaeyypa0JaeGymaeJaeyOeI0IaemiCaa3aaSbaaSqaaiabigdaXaqabaGccqaIXaqmcqaIWaamdaahaaWcbeqaaiabgkHiTiab=j7aIbaaaaaaaa@7CE6@

The combined cumulative distribution function is thus,

Fβ(β)={1−p110−βcβ=01−p110−ββ≥βc
 MathType@MTEF@5@5@+=feaafiart1ev1aaatCvAUfKttLearuWrP9MDH5MBPbIqV92AaeXatLxBI9gBaebbnrfifHhDYfgasaacH8akY=wiFfYdH8Gipec8Eeeu0xXdbba9frFj0=OqFfea0dXdd9vqai=hGuQ8kuc9pgc9s8qqaq=dirpe0xb9q8qiLsFr0=vr0=vr0dc8meaabaqaciaacaGaaeqabaqabeGadaaakeaacqWGgbGrdaWgaaWcbaacciGae8NSdigabeaakiabcIcaOiab=j7aIjabcMcaPiabg2da9maaceaabaqbaeaabiGaaaqaaiabigdaXiabgkHiTiabdchaWnaaBaaaleaacqaIXaqmaeqaaOGaeGymaeJaeGimaaZaaWbaaSqabeaacqGHsislcqWFYoGydaWgaaadbaGaem4yamgabeaaaaaakeaacqWFYoGycqGH9aqpcqaIWaamaeaacqaIXaqmcqGHsislcqWGWbaCdaWgaaWcbaGaeGymaedabeaakiabigdaXiabicdaWmaaCaaaleqabaGaeyOeI0Iae8NSdigaaaGcbaGae8NSdiMaeyyzImRae8NSdi2aaSbaaSqaaiabdogaJbqabaaaaaGccaGL7baaaaa@52BF@

When *β *≥ *β*_*c*_, *F*_*β*_(*β*) is continuous and the probability density function of pp value for random matches is

fβ(β)=ddβFβ(β)=ddβ(1−p110−β)=p1ln⁡(10)10−β(β≥βc)
 MathType@MTEF@5@5@+=feaafiart1ev1aaatCvAUfKttLearuWrP9MDH5MBPbIqV92AaeXatLxBI9gBaebbnrfifHhDYfgasaacH8akY=wiFfYdH8Gipec8Eeeu0xXdbba9frFj0=OqFfea0dXdd9vqai=hGuQ8kuc9pgc9s8qqaq=dirpe0xb9q8qiLsFr0=vr0=vr0dc8meaabaqaciaacaGaaeqabaqabeGadaaakeaafaqabeqacaaabaGaemOzay2aaSbaaSqaaGGaciab=j7aIbqabaGccqGGOaakcqWFYoGycqGGPaqkcqGH9aqpdaWcaaqaaiabdsgaKbqaaiabdsgaKjab=j7aIbaacqWGgbGrdaWgaaWcbaGae8NSdigabeaakiabcIcaOiab=j7aIjabcMcaPiabg2da9maalaaabaGaemizaqgabaGaemizaqMae8NSdigaaiabcIcaOiabigdaXiabgkHiTiabdchaWnaaBaaaleaacqaIXaqmaeqaaOGaeGymaeJaeGimaaZaaWbaaSqabeaacqGHsislcqWFYoGyaaGccqGGPaqkcqGH9aqpcqWGWbaCdaWgaaWcbaGaeGymaedabeaakiGbcYgaSjabc6gaUjabcIcaOiabigdaXiabicdaWiabcMcaPiabigdaXiabicdaWmaaCaaaleqabaGaeyOeI0Iae8NSdigaaaGcbaGaeiikaGIae8NSdiMaeyyzImRae8NSdi2aaSbaaSqaaiabdogaJbqabaGccqGGPaqkaaaaaa@6591@

### Confidence level for pp and pp2 values

The confidence level can also be determined for both pp and pp2. Suppose there are *r *theoretical spectra within the protein sequence database. If we assume that all theoretical spectra are uncorrelated, eqn. 37 gives *φ*, the number of random matches that have a pp value greater than or equal to *β *under H_0 _for any given experimental spectrum.

φ=r∫β+∞fβ(x)dx=r∫β+∞p1ln⁡(10)10−xdx=rp110−β,
 MathType@MTEF@5@5@+=feaafiart1ev1aaatCvAUfKttLearuWrP9MDH5MBPbIqV92AaeXatLxBI9gBaebbnrfifHhDYfgasaacH8akY=wiFfYdH8Gipec8Eeeu0xXdbba9frFj0=OqFfea0dXdd9vqai=hGuQ8kuc9pgc9s8qqaq=dirpe0xb9q8qiLsFr0=vr0=vr0dc8meaabaqaciaacaGaaeqabaqabeGadaaakeaaiiGacqWFgpGzcqGH9aqpcqWGYbGCdaWdXaqaaiabdAgaMnaaBaaaleaacqWFYoGyaeqaaOGaeiikaGIaemiEaGNaeiykaKIaemizaqMaemiEaGhaleaacqWFYoGyaeaacqGHRaWkcqGHEisPa0Gaey4kIipakiabg2da9iabdkhaYnaapedabaGaemiCaa3aaSbaaSqaaiabigdaXaqabaaabaGae8NSdigabaGaey4kaSIaeyOhIukaniabgUIiYdGccyGGSbaBcqGGUbGBcqGGOaakcqaIXaqmcqaIWaamcqGGPaqkcqaIXaqmcqaIWaamdaahaaWcbeqaaiabgkHiTiabdIha4baakiabdsgaKjabdIha4jabg2da9iabdkhaYjabdchaWnaaBaaaleaacqaIXaqmaeqaaOGaeGymaeJaeGimaaZaaWbaaSqabeaacqGHsislcqWFYoGyaaGccqGGSaalaaa@6347@

The confidence level, *ψ*, is defined as

*ψ *= -log(*φ*) = -log(*r p*_1 _10^-*β*^) = *β *-log(*r*)-log(*p*_1_)

where *β *is either the pp or pp2 value, *r *is the number of theoretical spectra within the protein sequence database, and *p*_1 _is given in eqn. 4. Confidence levels calculated from pp value and pp2 value are referred as confidence level and confidence level2 respectively.

The confidence level is the negative common logarithm of the expected number of random matches with a pp value bigger than or equal to the one we observe for the corresponding experimental spectrum. Therefore, if the confidence level is below 0, more than one random match for the spectrum is expected and the corresponding match is highly suspect. From eqn. 38, the pp value is directly related to the confidence value. The confidence level is dependent upon the size of the database and degrades as the number of peptide created from the database increases.

### The protein pp score

The pp model is also used to calculate pp values for protein matches. Let *r*_protein _denote the total number of theoretical spectra created from a protein sequence and *n*_spectra _denote the total number of experimental spectra in the data set. The cross match of all experimental spectra with theoretical peptides for the protein sequence generates *n*_match_protein _= *r*_protein _× *n*_spctra _potential matches. The sum of reported pp values of all matches for the protein is calculated from eqn. 39.

B=∑i=1nmatch_proteinβi
 MathType@MTEF@5@5@+=feaafiart1ev1aaatCvAUfKttLearuWrP9MDH5MBPbIqV92AaeXatLxBI9gBaebbnrfifHhDYfgasaacH8akY=wiFfYdH8Gipec8Eeeu0xXdbba9frFj0=OqFfea0dXdd9vqai=hGuQ8kuc9pgc9s8qqaq=dirpe0xb9q8qiLsFr0=vr0=vr0dc8meaabaqaciaacaGaaeqabaqabeGadaaakeaacqWGcbGqcqGH9aqpdaaeWbqaaGGaciab=j7aInaaBaaaleaacqWGPbqAaeqaaaqaaiabdMgaPjabg2da9iabigdaXaqaaiabd6gaUnaaBaaameaacqqGTbqBcqqGHbqycqqG0baDcqqGJbWycqqGObaAcqqGFbWxcqqGWbaCcqqGYbGCcqqGVbWBcqqG0baDcqqGLbqzcqqGPbqAcqqGUbGBaeqaaaqdcqGHris5aaaa@4ABD@

The statistical model is used to test the following hypotheses: H_0 _– All peptide matches for a given protein are random and H_A _– At least one peptide match for a given protein is not random. We assume that *r*_spectra _theoretical spectra created from the protein sequence are uncorrelated to each other and that *n*_spectra _experimental spectra from the data set are uncorrelated to each other. Since *n*_match_protein _is normally very large, *B *is approximately a normal distribution with a mean of *μ*_*B *_= *n*_match_protein _× *μ*_*β *_and a variance of *σ*_*B*_^2 ^= *n*_match_protein _× *σ*_*β*_^2 ^according to the central limit theorem.

According to the distribution of the pp value for random matches described above, the mean and variances of a random match are given by the following equations:

μβ=∫0+∞xfβ(x)dx=∫βc+∞xp1ln⁡(10)10−xdx=(p1ln⁡(10)+p1βc)10−βc
 MathType@MTEF@5@5@+=feaafiart1ev1aaatCvAUfKttLearuWrP9MDH5MBPbIqV92AaeXatLxBI9gBaebbnrfifHhDYfgasaacH8akY=wiFfYdH8Gipec8Eeeu0xXdbba9frFj0=OqFfea0dXdd9vqai=hGuQ8kuc9pgc9s8qqaq=dirpe0xb9q8qiLsFr0=vr0=vr0dc8meaabaqaciaacaGaaeqabaqabeGadaaakeaaiiGacqWF8oqBdaWgaaWcbaGae8NSdigabeaakiabg2da9maapedabaGaemiEaGNaemOzay2aaSbaaSqaaiab=j7aIbqabaGccqGGOaakcqWG4baEcqGGPaqkcqWGKbazcqWG4baEaSqaaiabicdaWaqaaiabgUcaRiabg6HiLcqdcqGHRiI8aOGaeyypa0Zaa8qmaeaacqWG4baEcqWGWbaCdaWgaaWcbaGaeGymaedabeaakiGbcYgaSjabc6gaUjabcIcaOiabigdaXiabicdaWiabcMcaPiabigdaXiabicdaWmaaCaaaleqabaGaeyOeI0IaemiEaGhaaOGaemizaqMaemiEaGhaleaacqWFYoGydaWgaaadbaGaem4yamgabeaaaSqaaiabgUcaRiabg6HiLcqdcqGHRiI8aOGaeyypa0JaeiikaGYaaSaaaeaacqWGWbaCdaWgaaWcbaGaeGymaedabeaaaOqaaiGbcYgaSjabc6gaUjabcIcaOiabigdaXiabicdaWiabcMcaPaaacqGHRaWkcqWGWbaCdaWgaaWcbaGaeGymaedabeaakiab=j7aInaaBaaaleaacqWGJbWyaeqaaOGaeiykaKIaeGymaeJaeGimaaZaaWbaaSqabeaacqGHsislcqWFYoGydaWgaaadbaGaem4yamgabeaaaaaaaa@73EB@

and

σβ2=E(β2)−μβ2=∫0+∞x2fβ(x)dx−μβ2=∫βc+∞x2p1ln⁡(10)10−xdx−μβ2=(2p1[ln⁡(10)]2+2p1βcln⁡(10)+p1βc 2)10−βc−[(p1ln⁡(10)+p1βc)10−βc]2
 MathType@MTEF@5@5@+=feaafiart1ev1aaatCvAUfKttLearuWrP9MDH5MBPbIqV92AaeXatLxBI9gBamXvP5wqSXMqHnxAJn0BKvguHDwzZbqegyvzYrwyUfgarqqtubsr4rNCHbGeaGqiA8vkIkVAFgIELiFeLkFeLk=iY=Hhbbf9v8qqaqFr0xc9pk0xbba9q8WqFfeaY=biLkVcLq=JHqVepeea0=as0db9vqpepesP0xe9Fve9Fve9GapdbaqaaeGacaGaaiaabeqaamqadiabaaGcbaqbaeWabiqaaaqaaGGaciab=n8aZnaaDaaaleaacqWFYoGyaeaacqaIYaGmaaGccqGH9aqpcqWGfbqrcqGGOaakcqWFYoGydaahaaWcbeqaaiabikdaYaaakiabcMcaPiabgkHiTiab=X7aTnaaDaaaleaacqWFYoGyaeaacqaIYaGmaaGccqGH9aqpdaWdXaqaaiabdIha4naaCaaaleqabaGaeGOmaidaaOGaemOzay2aaSbaaSqaaiab=j7aIbqabaGccqGGOaakcqWG4baEcqGGPaqkcqWGKbazcqWG4baEaSqaaiabicdaWaqaaiabgUcaRiabg6HiLcqdcqGHRiI8aOGaeyOeI0Iae8hVd02aa0baaSqaaiab=j7aIbqaaiabikdaYaaakiabg2da9maapedabaGaemiEaG3aaWbaaSqabeaacqaIYaGmaaGccqWGWbaCdaWgaaWcbaGaeGymaedabeaakiGbcYgaSjabc6gaUjabcIcaOiabigdaXiabicdaWiabcMcaPiabigdaXiabicdaWmaaCaaaleqabaGaeyOeI0IaemiEaGhaaOGaemizaqMaemiEaGhaleaacqWFYoGydaWgaaadbaGaem4yamgabeaaaSqaaiabgUcaRiabg6HiLcqdcqGHRiI8aOGaeyOeI0Iae8hVd02aa0baaSqaaiab=j7aIbqaaiabikdaYaaaaOqaaiabg2da9iabcIcaOmaalaaabaGaeGOmaiJaemiCaa3aaSbaaSqaaiabigdaXaqabaaakeaadaWadaqaaiGbcYgaSjabc6gaUjabcIcaOiabigdaXiabicdaWiabcMcaPaGaay5waiaaw2faamaaCaaaleqabaGaeGOmaidaaaaakiabgUcaRmaalaaabaGaeGOmaiJaemiCaa3aaSbaaSqaaiabigdaXaqabaGccqWFYoGydaWgaaWcbaGaem4yamgabeaaaOqaaiGbcYgaSjabc6gaUjabcIcaOiabigdaXiabicdaWiabcMcaPaaacqGHRaWkcqWGWbaCdaWgaaWcbaGaeGymaedabeaakiab=j7aInaaDaaaleaacqqGJbWyaeaacqqGGaaicqqGYaGmaaGccqGGPaqkcqaIXaqmcqaIWaamdaahaaWcbeqaaiabgkHiTiab=j7aInaaBaaameaacqWGJbWyaeqaaaaakiabgkHiTmaadmaabaGaeiikaGYaaSaaaeaacqWGWbaCdaWgaaWcbaGaeGymaedabeaaaOqaaiGbcYgaSjabc6gaUjabcIcaOiabigdaXiabicdaWiabcMcaPaaacqGHRaWkcqWGWbaCdaWgaaWcbaGaeGymaedabeaakiabek7aInaaBaaaleaacqWGJbWyaeqaaOGaeiykaKIaeGymaeJaeGimaaZaaWbaaSqabeaacqGHsislcqWFYoGydaWgaaadbaGaem4yamgabeaaaaaakiaawUfacaGLDbaadaahaaWcbeqaaiabikdaYaaaaaaaaa@CD4C@

where *p*_1 _is given in eqn. 4 and *β*_*c *_is the pp value threshold. Likewise for the sum of pp values for the protein, *B*, the mean and variance for the distribution under H_0 _are given in eqn. 42:

{μB=nmatch_protein(p1ln⁡(10)+p1βc)10−βcσB2=nmatch_protein{[2p1[ln⁡(10)]2+2p1βcln⁡(10)+p1βc 2]10−βc−[(p1ln⁡(10)+p1βc)10−βc]2}
 MathType@MTEF@5@5@+=feaafiart1ev1aaatCvAUfKttLearuWrP9MDH5MBPbIqV92AaeXatLxBI9gBamXvP5wqSXMqHnxAJn0BKvguHDwzZbqegyvzYrwyUfgarqqtubsr4rNCHbGeaGqiA8vkIkVAFgIELiFeLkFeLk=iY=Hhbbf9v8qqaqFr0xc9pk0xbba9q8WqFfeaY=biLkVcLq=JHqVepeea0=as0db9vqpepesP0xe9Fve9Fve9GapdbaqaaeGacaGaaiaabeqaamqadiabaaGcbaWaaiqaaeaafaqaaeGabaaabaacciGae8hVd02aaSbaaSqaaiabdkeacbqabaGccqGH9aqpcqWGUbGBdaWgaaWcbaGaeeyBa0MaeeyyaeMaeeiDaqNaee4yamMaeeiAaGMaee4xa8LaeeiCaaNaeeOCaiNaee4Ba8MaeeiDaqNaeeyzauMaeeyAaKMaeeOBa4gabeaakiabcIcaOmaalaaabaGaemiCaa3aaSbaaSqaaiabigdaXaqabaaakeaacyGGSbaBcqGGUbGBcqGGOaakcqaIXaqmcqaIWaamcqGGPaqkaaGaey4kaSIaemiCaa3aaSbaaSqaaiabigdaXaqabaGccqWFYoGydaWgaaWcbaGaem4yamgabeaakiabcMcaPiabigdaXiabicdaWmaaCaaaleqabaGaeyOeI0Iae8NSdi2aaSbaaWqaaiabdogaJbqabaaaaaGcbaGae83Wdm3aa0baaSqaaiabdkeacbqaaiabikdaYaaakiabg2da9iabd6gaUnaaBaaaleaacqqGTbqBcqqGHbqycqqG0baDcqqGJbWycqqGObaAcqqGFbWxcqqGWbaCcqqGYbGCcqqGVbWBcqqG0baDcqqGLbqzcqqGPbqAcqqGUbGBaeqaaOWaaiWaaeaadaWadaqaamaalaaabaGaeGOmaiJaemiCaa3aaSbaaSqaaiabigdaXaqabaaakeaadaWadaqaaiGbcYgaSjabc6gaUjabcIcaOiabigdaXiabicdaWiabcMcaPaGaay5waiaaw2faamaaCaaaleqabaGaeGOmaidaaaaakiabgUcaRmaalaaabaGaeGOmaiJaemiCaa3aaSbaaSqaaiabigdaXaqabaGccqWFYoGydaWgaaWcbaGaem4yamgabeaaaOqaaiGbcYgaSjabc6gaUjabcIcaOiabigdaXiabicdaWiabcMcaPaaacqGHRaWkcqWGWbaCdaWgaaWcbaGaeGymaedabeaakiab=j7aInaaDaaaleaacqqGJbWyaeaacqqGGaaicqqGYaGmaaaakiaawUfacaGLDbaacqaIXaqmcqaIWaamdaahaaWcbeqaaiabgkHiTiab=j7aInaaBaaameaacqWGJbWyaeqaaaaakiabgkHiTmaadmaabaGaeiikaGYaaSaaaeaacqWGWbaCdaWgaaWcbaGaeGymaedabeaaaOqaaiGbcYgaSjabc6gaUjabcIcaOiabigdaXiabicdaWiabcMcaPaaacqGHRaWkcqWGWbaCdaWgaaWcbaGaeGymaedabeaakiab=j7aInaaBaaaleaacqWGJbWyaeqaaOGaeiykaKIaeGymaeJaeGimaaZaaWbaaSqabeaacqGHsislcqWFYoGydaWgaaadbaGaem4yamgabeaaaaaakiaawUfacaGLDbaadaahaaWcbeqaaiabikdaYaaaaOGaay5Eaiaaw2haaaaaaiaawUhaaaaa@CDCB@

The p-value for a protein, *α*_protein_, is defined to be the probability that the protein hit can have a sum of pp values from all its peptide matches greater than or equal to *B *under H_0. _Thus *α*_protein _is given by

αprotein=∫B+∞fB(x)dx=∫B+∞e−(x−μB)2/(2σB2)2πσBdx.
 MathType@MTEF@5@5@+=feaafiart1ev1aaatCvAUfKttLearuWrP9MDH5MBPbIqV92AaeXatLxBI9gBaebbnrfifHhDYfgasaacH8akY=wiFfYdH8Gipec8Eeeu0xXdbba9frFj0=OqFfea0dXdd9vqai=hGuQ8kuc9pgc9s8qqaq=dirpe0xb9q8qiLsFr0=vr0=vr0dc8meaabaqaciaacaGaaeqabaqabeGadaaakeaaiiGacqWFXoqydaWgaaWcbaGaeeiCaaNaeeOCaiNaee4Ba8MaeeiDaqNaeeyzauMaeeyAaKMaeeOBa4gabeaakiabg2da9maapedabaGaemOzay2aaSbaaSqaaiabdkeacbqabaGccqGGOaakcqWG4baEcqGGPaqkcqWGKbazcqWG4baEaSqaaiabdkeacbqaaiabgUcaRiabg6HiLcqdcqGHRiI8aOGaeyypa0Zaa8qmaeaadaWcaaqaaiabdwgaLnaaCaaaleqabaGaeyOeI0IaeiikaGIaemiEaGNaeyOeI0Iae8hVd02aaSbaaWqaaiabdkeacbqabaWccqGGPaqkdaahaaadbeqaaiabikdaYaaaliabc+caViabcIcaOiabikdaYiab=n8aZnaaDaaameaacqWGcbGqaeaacqaIYaGmaaWccqGGPaqkaaaakeaadaGcaaqaaiabikdaYiab=b8aWbWcbeaakiab=n8aZnaaBaaaleaacqWGcbGqaeqaaaaakiabdsgaKjabdIha4bWcbaGaemOqaieabaGaey4kaSIaeyOhIukaniabgUIiYdGccqGGUaGlaaa@69B8@

and the protein pp value becomes

protein pp value=−log⁡(αprotein)=−log⁡(∫B+∞e−(x−μB)2/(2σB2)2πσBdx)
 MathType@MTEF@5@5@+=feaafiart1ev1aaatCvAUfKttLearuWrP9MDH5MBPbIqV92AaeXatLxBI9gBaebbnrfifHhDYfgasaacH8akY=wiFfYdH8Gipec8Eeeu0xXdbba9frFj0=OqFfea0dXdd9vqai=hGuQ8kuc9pgc9s8qqaq=dirpe0xb9q8qiLsFr0=vr0=vr0dc8meaabaqaciaacaGaaeqabaqabeGadaaakeaacqqGWbaCcqqGYbGCcqqGVbWBcqqG0baDcqqGLbqzcqqGPbqAcqqGUbGBcqqGGaaicqqGWbaCcqqGWbaCcqqGGaaicqqG2bGDcqqGHbqycqqGSbaBcqqG1bqDcqqGLbqzcqGH9aqpcqGHsislcyGGSbaBcqGGVbWBcqGGNbWzcqGGOaakiiGacqWFXoqydaWgaaWcbaGaeeiCaaNaeeOCaiNaee4Ba8MaeeiDaqNaeeyzauMaeeyAaKMaeeOBa4gabeaakiabcMcaPiabg2da9iabgkHiTiGbcYgaSjabc+gaVjabcEgaNjabcIcaOmaapedabaWaaSaaaeaacqWGLbqzdaahaaWcbeqaaiabgkHiTiabcIcaOiabdIha4jabgkHiTiab=X7aTnaaBaaameaacqWGcbGqaeqaaSGaeiykaKYaaWbaaWqabeaacqaIYaGmaaWccqGGVaWlcqGGOaakcqaIYaGmcqWFdpWCdaqhaaadbaGaemOqaieabaGaeGOmaidaaSGaeiykaKcaaaGcbaWaaOaaaeaacqaIYaGmcqWFapaCaSqabaGccqWFdpWCdaWgaaWcbaGaemOqaieabeaaaaGccqWGKbazcqWG4baEaSqaaiabdkeacbqaaiabgUcaRiabg6HiLcqdcqGHRiI8aOGaeiykaKcaaa@7D1A@

## Discussion

### Effect of various spectral characteristics on scoring

**Figure 1:**
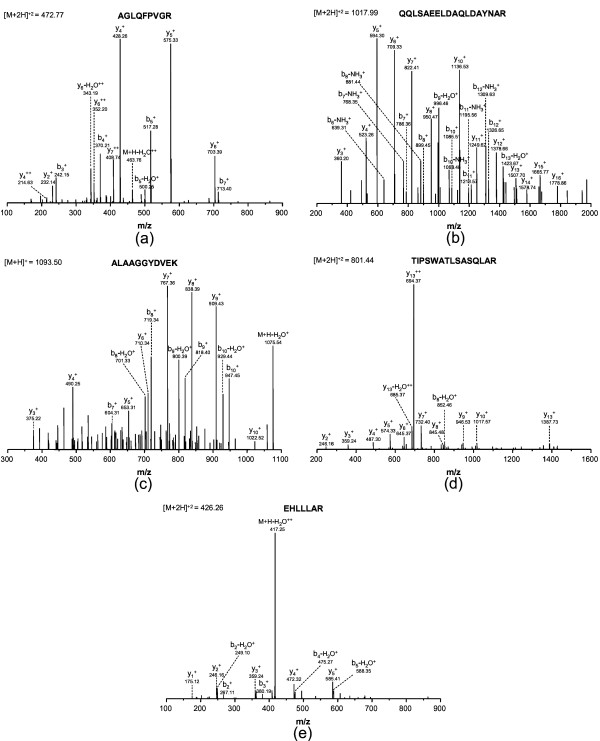
Examples of spectral influences on scoring. High quality data for peptides of short (a) and long (b) lengths. Poorer quality data from a low signal-to-noise spectrum (c), a spectrum from a peptide with a single dominant product ion due to fragmentation at the N-terminal side of Pro (d) and a spectrum of a peptide with a single dominant product ion due to a extensive neutral loss of water at the N-terminal Glu (e) [30].

Five example spectra, shown in Figure 1, are used to illustrate the effect of various spectral characteristics on scoring. All spectra were collected on an LTQ-Orbitrap mass spectrometer (ThermoElectron Finnigan, San Jose, CA, USA) [29]. Precursor and product ions were mass analyzed by the Orbitrap to achieve a mass accuracy of < 5.0 ppm. The pp and pp2 values at different mass accuracies (0.01 Da, 0.1 Da and 1.0 Da) were calculated and listed in Table 1. Mass accuracy tolerances were specified as either relative or absolute for precursor ions but only as absolute tolerances for product ions. Absolute mass accuracy tolerances for product ions are computationally cheaper and yield a reasonable compromise between computational expense and accuracy. Good quality spectra (Figure 1a,1b) yielded high empirical and statistical scores as expected. These sequences in Figure 1a &1b illustrate that peptide length has little effect on the scoring. This observation is consistent with the statistical model lack of peptide sequence bias and the peptide length penalty included in the empirical score (eqn. 1). Low quality data (i.e. low signal to noise ratio) can still yield good scores if the most abundant ions are dominated by signal (Figure 1c). The most challenging spectra are those with few dominant signal peaks. Examples are shown in 1d &1e. These figures show the spectra with one single dominant ion due to N-terminal fragmentation of an internal Pro residue and the neutral loss of H_2_O at an N-terminal Glu residue [30]. The empirical scores were poorer for these cases since only a single ion mainly contributes to score (eqn. 1). However, the pp and pp2 values were not as severely affected and able to accurately discriminate these matches from false positives.

**Table 1:** Empirical and statistical scores along with associated parameters (*p*1 and *p*2) for each spectrum shown in Figure 1. The data were obtained for mass accuracies of 1.0 Da, 0.1 Da and 0.01 Da. Confidence levels were calculated based on a search space of 2726345 theoretical peptides. The confidence levels for the pp and pp2 scores are denotes as confidence level and confidence level2

**Mass accuracy**	**Spectrum**	***p*1**	***p*2**	**score**	**pp**	**pp2**	**Confidence level**	**Confidence level 2**
0.01 Da	a	2.1. × 10^-5^	7.9. × 10^-4^	69	53.8	307.6	52.0	305.8
	b	2.1. × 10^-5^	2.1. × 10^-3^	75	69.0	307.6	67.2	305.8
	c	2.1. × 10^-5^	9.2. × 10^-4^	59	43.0	307.6	41.2	305.8
	d	2.1. × 10^-5^	1.5. × 10^-3^	19	75.2	307.6	73.4	305.8
	e	2.1. × 10^-5^	6.5. × 10^-4^	27	36.6	307.6	34.8	305.8

0.1 Da	a	2.1. × 10^-4^	7.8. × 10^-3^	77	38.6	203.9	35.8	201.1
	b	2.1. × 10^-4^	2.1. × 10^-2^	95	46.7	157.5	43.9	154.7
	c	2.1. × 10^-4^	9.1. × 10^-3^	68	26.9	274.7	24.1	271.9
	d	2.1. × 10^-4^	1.5. × 10^-2^	20	45.2	37.3	42.4	34.5
	e	2.1. × 10^-4^	6.5. × 10^-3^	28	23.7	82.8	20.9	80.0

1.0 Da	a	2.1. × 10^-3^	7.8. × 10^-2^	100	22.1	21.3	18.3	17.5
	b	2.1. × 10^-3^	2.1. × 10^-1^	120	16.0	12.1	12.2	8.3
	c	2.1. × 10^-3^	9.1. × 10^-2^	74	7.8	23.1	4.0	19.3
	d	2.1. × 10^-3^	1.5. × 10^-1^	55	15.5	6.1	11.7	2.3
	e	2.1. × 10^-3^	6.4. × 10^-2^	36	14.7	9.0	10.9	5.2

### Comparison between pp and pp2 values

The pp value is the primary discriminator for quality of matches. The pp2 value can provide a complementary assessment of quality when pp values are suspect. Although the pp and pp2 value have the same statistical basis, there are several differences between them: The pp value is based on the number of matched product ions and the pp2 value is based on the total abundance of matched product ions. The pp value can be underestimated when noise is present in the experimental spectrum especially at low mass accuracy. Because noise normally has lower abundance than product ions, the pp2 value, on the other hand, is generally unaffected. As shown in Table 1, pp value for the spectrum with low signal to noise ratio and majority of noise peaks (Figure 1c) were affected negatively by the noise peaks and relative low compared with those for normal spectra at mass accuracy of 1.0 Da. However, pp2 value was not affected by the noise peaks.

While pp value can be precisely calculated, there are three assumptions needed to estimate the pp2 values. Assumption 1 and 3 for the pp2 test are not plausible when the number of product ions in the experimental spectrum, *n *is small. Therefore, pp2 value estimated by the central limit theorem cannot evaluate the quality of matches with a small number of product ions. Furthermore the normal distribution under the central limit theorem is less tailed than the true distribution of *Y*, pp2 value is normally overestimated when it is large (> 16) as shown in Table 1.

From the above discussion, the pp value is more reliable and accurate than the pp2 value under most circumstances, but it can be affected by noise. Under these circumstances, the number of product ions in the spectrum is normally large and pp2 value can be well estimated and complementary to pp value. Thus the combination of the two scores provides an excellent means to ascertain the quality of matches under conditions where one might fail.

### Effect of mass accuracy on pp values

In the pp model, the two most important parameters (*p*_1_, the probability that a theoretical precursor randomly matches the experimental and *p*_2_, the probability that a theoretical product ion randomly matches any product ions in the MS/MS spectrum) are set in accordance with the predetermined mass accuracy of mass spectrometer. These parameters' values decrease as mass accuracy increases. This effect is shown in Table 1. A more thorough list of all parameters used in calculating the empirical and statistical scores is provided as supplementary material [see Additional file 1]. The statistical model specifically takes each parameter into account when calculating the statistical scores. Therefore, these two parameters have a substantial effect on the pp values for both random matches and true matches. Consequently, pp and pp2 values are very sensitive to the accuracy of mass spectrometer.

**Figure 2:**
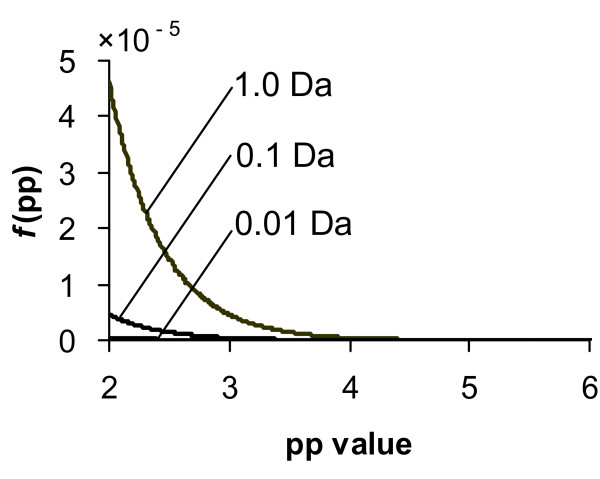
Effect of mass accuracy on the theoretical probability density function (*f*(pp)) of pp and pp2 values for random matches (*β *≥ *β*_*c*_).

As is shown in the Figure 2, the probability of random matches having high pp values is substantially reduced as we increase mass accuracy. Increasing mass accuracy resulted in a shift of the pp value distribution for random matches to lower values. At the same time the pp value distribution for true matches moves to higher pp values. This effect is evident from the pp values in Table 1. As mass accuracy improved, the pp values improved for all peptide matches in Figure 1. Thus higher mass accuracy improves sensitivity and selectivity for a search and help discriminate true matches from random matches.

## Conclusion

A new statistically derived scoring algorithm was developed for characterization of peptides, proteins and their posttranslational modifications from tandem MS data. The probability based algorithm implicitly incorporates mass accuracy into scoring the potential peptide and protein matches. This approach is separate and distinct from algorithms that filter precursor and product ion matches based on mass accuracy. The statistical model involves no empirical parameters and its scores correlate to the probability that a match is a random occurrence. A novel statistically derived algorithm to rigorously calculate protein scores from the probability based peptide scores was also developed. Thus the protein scores reflect the significance of protein matches and can be used to differentiate true protein matches from random matches. The algorithm is incorporated in an automated database search program MassMatrix.

## Authors' contributions

HX designed and mathematically proved the statistical model and drafted the manuscript. MAF was the principle investigator and provided overall guidance of the project, and also revised the manuscript critically. Both authors read and approved the final manuscript.
